# OATP1B1 Plays an Important Role in the Transport and Treatment Efficacy of Sorafenib in Hepatocellular Carcinoma

**DOI:** 10.1155/2021/9711179

**Published:** 2021-09-26

**Authors:** Jinhua Wen, Menghua Zhao

**Affiliations:** ^1^Department of GCP, the First Affiliated Hospital of Nanchang University, Nanchang 330006, China; ^2^School of Pharmacy, Nanchang University, Nanchang 330006, China

## Abstract

**Background:**

Sorafenib is an anticancer drug used in the treatment of unresectable hepatocellular carcinoma and advanced renal cell carcinoma. It is a substrate for the human OATP1B1. This study is aimed at assessing the role of OATP1B1 in transportation and uptake of sorafenib in hepatocellular carcinoma and how OATP1B1 affects the pharmacodynamics of sorafenib in vitro and in vivo.

**Methods:**

Sorafenib transport was measured in HepG2, HepG2-OATP1B1∗1a, HepG2-OATP1B1∗1b, HepG2-OATP1B1∗15, LO2, LO2-OATP1B1∗1a, LO2-OATP1B1∗1b, and LO2-OATP1B1∗15 cells, as well as in HepG2 cells transfected with miR-148a mimics. The viability and apoptosis rate of cells treated with sorafenib were evaluated. A liver cancer rat model was established to explore the pharmacokinetics and pharmacodynamics of sorafenib after overexpression of Oatp2.

**Results:**

Changes in expression and genetic mutations of OATP1B1 significantly affected the uptake of sorafenib in HepG2 and LO2 transgenic cells, and the uptake of sorafenib was higher in HepG2 than LO2. Genetic mutations of OATP1B1 significantly affected the cell viability and apoptosis rate of HepG2 cells after sorafenib treatment. Compared to control group, the uptake of sorafenib in miR-148a mimic-transfected HepG2 cells was decreased, and the cell viability was increased. PCN significantly increased the expression of Oatp2 and affected the pharmacokinetics of sorafenib. Vascular endothelial growth factor levels and microvascular density in tumor-adjacent tissues decreased significantly, suggesting that increased Oatp2 expression improves the treatment effect of sorafenib in a rat model of liver cancer.

**Conclusions:**

OATP1B1 plays an important role in the pharmacokinetics and pharmacodynamics of sorafenib in hepatocellular carcinoma.

## 1. Background

Sorafenib is approved by the United States Food and Drug Administration for the treatment of unresectable hepatocellular carcinoma and advanced renal cell carcinoma. Sorafenib inhibits tumor growth and angiogenesis by targeting both the RAF/MEK/ERK pathway and receptor tyrosine kinases [1]. In humans, sorafenib is administered in tablet form, and the majority (77%) of the sorafenib dose is either unabsorbed or eliminated through the hepatobiliary route (50% unchanged), and 19% of the dose (mostly glucuronides) is excreted in urine [2]. The liver is the main target organ of sorafenib. A study using a HEK293 cell model demonstrated that sorafenib was a substrate for the human organic anion transport polypeptide 1B1 (OATP1B1) and caused a dramatic increase in plasma levels of sorafenib-glucuronide [3]. However, the sorafenib transportation mediated by OATP1B1 and its effect on cancer are not clear. The expression and function of OATPs also change under conditions of liver cancer. OATP1B1 (also known as OATP-C or LST-1 and coded by the gene *SLCO1B1*) is an uptake transporter expressed in the basolateral (sinusoidal) membrane of hepatocytes and plays an important role in the transport of endogenous substances and a variety of clinical drugs [4]. It has 2 single nucleotide polymorphisms: 388 A > G (63% mutation frequency in Asian populations) and 521 T > C (16% mutation frequency in Asian populations) [5], which form four haplotypes: OATP1B1∗1a (c.388APc.521 T), OATP1B1∗1b (c.388GPc.521 T), OABP1B1∗5 (c.388APc.521 C), and OATP1B1∗15 (c.388GPc.521 C). Many studies have revealed that genetic mutations affected OATP1B1 transporter activity and exert a significant effect on drug transport [6]. Genetic polymorphisms could also significantly alter the transporting activities of OATP1B1 [7]. Therefore, OATP1B1 plays an important role in drug transport and clinical treatment. However, its expression, function, and effect on drug treatment in hepatocellular carcinoma tissue are not known. It is also unclear whether genetic mutations of OATP1B1 have different effects on OATP1B1-mediated drug transport or cell viability in HepG2 cells. This study is the first to explore the effects of OATP1B1 mutation on drug transportation and its effects on tumor suppression. It has been reported that miR-148a can regulate the expression of OATP1B1 meditated by PXR [8], which played a key role in regulating the expression of some metabolic enzymes and relevant drug transporters [9]. Therefore, we also investigated the effects of miR-148a mimics on the expression of the OATP1B1 gene in HepG2 cells, as well as the functions of OATP1B1 in tumor treatment. Finally, we upregulated the expression of Oatp2 in a rat model of hepatocellular carcinoma to explore the changes in drug pharmacokinetics and the therapeutic effects of Oatp2 on hepatocellular carcinoma. Our experiments are aimed at elucidating the role of OATP1B1 in the pharmacokinetics of sorafenib treatment in hepatocellular carcinoma.

## 2. Experimental Materials and Methods

### 2.1. Materials and Main Instruments

The reagents used were as follows: HepG2, LO2, and lentivirus are purchased from Hangzhou Hibio (Hangzhou, China); sorafenib (>99% purity), China Science & Technology Co., Ltd. (Hangzhou, China); Pregnenolone-16*α*-carbonitrile (PCN), Cayman Chemical (Ann Arbor, MI, USA); OATP1B1 antibody, Abcam (Cambridge, UK); goat anti-mouse IgG (GAM007), goat anti-rabbit IgG (GAR007), and glyceraldehyde-3-phosphate dehydrogenase (GAPDH), Allied biology; GAPDH (Mab5465), Lianke Biology, China; miR-148a mimics, Ribobio, Guangzhou, China; methanol and acetonitrile (chromatographic purity), Sigma-Aldrich (St. Louis, MO, USA); high purity RNA rapid extraction kit, fetal bovine serum, and BCA protein assay kit, Thermo Fisher Scientific Co., Ltd. (Massachusetts, USA); Hiscript II Q RT SuperMix for qPCR and ChamQ SYBR color qPCR master mix, Vazyme Biotech Co., Ltd. (Nanjing, China); SuperSignal West Dura, Thermo Fisher Scientific (Waltham, MA, USA); PrimeScript™ RT reagent kit, Takara Bio Inc. (Kusatsu, Shiga, Japan); and TransIntro™ EL Transfection Reagent, Transcript First-strand cDNA synthesis superMix, and TransStart™ Green qPCRSuperMix from TransGen (Beijing, China). The following instruments were also used: Mini-PROTEAN Tetra electrophoresis system and the ChemiDoc XRS+ gel imaging system, Bio-Rad (Hercules, CA, USA); a low-light spectrophotometer, Merinton Company (Beijing, China); flow cytometer, Becton, Dickinson and Company (Franklin Lakes, NJ, USA); cell incubator, Thermo Fisher Scientific; inverted microscope, Olympus (Shinjuku City, Tokyo, Japan); low-speed desktop centrifuge, Shanghai Medical Equipment Co., Ltd. (Shanghai, China); full-wavelength microplate reader, Molecular Devices, (SpectraMax i3x, Silicon Valley, USA); desktop high-speed refrigerated centrifuge, Xiangyi Instrument Co. Ltd. (Hunan, China); and high-performance liquid chromatography (HPLC) system, Shimadzu (LC-20AT, Shimadzu, Kyoto, Japan).

### 2.2. Animals

Male Sprague-Dawley rats (Shanghai slake experimental animal Co., Ltd., 20170005034834) aged approximately 100 days and weighing 250 ± 20 g were used in the experiments. Animal dosing procedures were performed in accordance with the ethical guidelines described in the Principles of Laboratory Animal Care (HB2019000003022).

### 2.3. Method

#### 2.3.1. Effect of OATP1B1 Genetic Mutations on Drug Transport and Treatment Effects in HepG2 and LO2 Cells

*(1) Cell Culture of HepG2 and LO2 Cells*. Based on the culture conditions and methods followed in previous studies, the hepatoma cell line HepG2 and the normal human hepatocyte cell line LO2 were cultured in an incubator (37°C, 5% CO_2_, and saturated humidity) in minimal essential medium containing 10% fetal bovine serum.

*(2) Establishment of HepG2-OATP1B1 and LO2-OATP1B1 Transgenic Cell Models*. In order to construct the plasmid target gene, the target genes of OATP1B1∗1a, OATP1B1∗1b, and OATP1B1∗15 were obtained using the following forward (F) and reverse (R) primers: OATP1B1∗1a, 5′-GGGGTACCATCATGGACCAAAATCAAC-3′ (F) and 5′-CTCGAGTGGAAACACAGAAGCAGAAG-3′ (R); OATP1B1∗1b, 5′-CTAAAGAAACTAATATCGATTCATCAGAAAATTC-3′ (F) and 5′-GAATTTTCTGATGAATCGATATTAGTTTCTTTAG-3′ (R); and OATP1B1∗15, 5′-CATGTGGATATATGCGTTCATGGGTAATATGC-3′ (F) and 5′-GCATATTACCCATGAACGCATATATCCACATG-3′ (R). HepG2 and LO2 transgenic cells expressing OATP1B1∗1a, OATP1B1∗1b, and OATP1B1∗15 genotypes were constructed using lentivirus technology. The pGC-FU GFP lentiviral vector transfer system was used as the gene transmission medium to construct the recombinant lentiviral vectors of OATP1B1∗1a, OATP1B1∗1b, and OATP1B1∗15-GFP fusion genes. Gene expression was detected using RT-qPCR testing and Western Blotting. Cell extracts were prepared in lysis buffer. The cell debris was removed by centrifugation at 12,000 × g at 4°C for 15 min, and the total protein concentration was measured using a BCA protein assay kit. Protein samples (50 *μ*g) were subjected to sodium dodecyl sulfate polyacrylamide gel electrophoresis (SDS-PAGE) and electrophoretically transferred to polyvinylidene difluoride (PVDF) membranes. Immunoblots were probed using the rabbit polyclonal OATP1B1 antibody (diluted 1 : 2000) with mouse polyclonal anti-*β*-actin (diluted 1 : 5000) antibody as the loading control. After incubation with horseradish peroxidase- (HRP-) conjugated secondary antibody, signals were detected by SuperSignal West Dura using a Bio-Rad ChemiDoc XRS imaging system, and densitometry analysis was performed using the Image Lab Software (Bio-Rad).

*(3) Uptake of Sorafenib in HepG2-OATP1B1 and LO2-OATP1B1 Cells*. The effect of OATP1B1 gene mutations on the transport of sorafenib in hepatoma cells was evaluated as follows: HepG2, LO-2, and corresponding virus-infected cells in the logarithmic growth phase were collected and diluted with culture medium to a concentration of 1.0 × 10^6^ cells/mL, added to a 12-well culture plate (0.5 mL per well), and cultured for 3 days. Two hours before the experiment, the old culture medium was slowly removed, and the cells were washed thrice using preheated uptake buffer solution. Following the final incubation at 37°C for 10 min, the uptake buffer was slowly removed at regular intervals, and uptake buffers containing different sorafenib concentrations (5, 10, and 15 *μ*M) were added. The cells were incubated at 37°C for 10 min, and the upper layer of the cells was slowly removed. After washing four times using 4°C uptake buffer, 0.2 mL sterile water was added for 3 freeze-thaw cycles in a -80°C ultralow temperature refrigerator. The cell lysate was transferred to an Eppendorf tube and centrifuged at 15,000 rpm for 10 min. The sorafenib content of cells was determined using high performance liquid chromatography (HPLC), and the protein content was determined through the Coomassie brilliant blue staining method. The cells in the experiments were divided into the following groups: HepG2 (control), HepG2-OATP1B1∗1a, HepG2-OATP1B1∗1b, HepG2-OATP1B1∗15, LO2 (control), LO2-OATP1B1∗1a, LO2-OATP1B1∗1b, and LO2-OATP1B1∗15.

*(4) Determination of Sorafenib Concentration*. HPLC-UV was used to determine the concentration of sorafenib. The mobile phase was acetonitrile/water/0.1% trifluoroacetic acid (45/35/20, *v*/*v*); flow rate, 1.0 mL/mL/min; column temperature, 35°C; UV detection wavelength, 266 nm; and injection volume, 20 *μ*L. The samples were extracted using acetonitrile.

*(5) Effect of OATP1B1 Genetic Mutation on Treatment Effect in HepG2 Cells*. To evaluate the effect of different OATP1B1 genotypes on tumor inhibition by sorafenib, the cell counting kit- (CCK-) 8 method was used to detect the proliferation of HepG2 cells, while flow cytometry was used to detect apoptosis in HepG2, HepG2-OATP1B1∗1a, HepG2-OATP1B1∗1b, and HepG2-OATP1B1∗15 cells.

#### 2.3.2. Effect of Regulating OATP1B1 Expression on the Viability and Apoptosis Rate of HepG2 Cell

*(1) Regulating OATP1B1 Expression in HepG2 Cells*. To investigate the effect of miR-148a on pregnane X receptor (PXR) and OATP1B1 expression, after miR-148a mimics were transfected into HepG2 cells with the TransIntro™ ELTransfection Reagent, mRNA expression levels of PXR and OATP1B1 were detected by Real-Time Quantitative reverse transcription PCR (RT-qPCR) testing and Western Blotting, respectively. Total RNA was extracted using an RNA extraction kit as per the manufacturer's instructions. RNA (2.0 *μ*g) was first reverse-transcribed to cDNA using the Transcriptor First-strand cDNA Synthesis Kit, and RT-qPCR was performed using TransStart™ Green qPCR SuperMix as per the manufacturer's instructions. The following primers were used: OATP1B1, 5′-AACTCCTACTGATTCTCGATGGG-3′ (F) and 5′-GTTTCCAGCACATGCAAAGAC-3′ (R); PXR, 5′-TTGCCCATCGAGGACCAGAT-3′ (F) and 5′-GTCTCCGCGTTGAACACTGT-3′ (R); and GAPDH, 5′-AGAAGGCTGGGGCTCATTTG-3′ (F) and 5′-AGGGGCCATCCACAGTCTTC-3′ (R). For Western Blotting, as previously reported [10], the total protein was first lysed with radio-immunoprecipitation assay buffer, and then, the protein concentrations were quantified using a BCA protein assay kit. Next, the protein samples (40 *μ*g) were separated using 10% SDS-PAGE and transferred onto a PVDF membrane. Subsequently, the PVDF membranes were blocked for 2 h with 5% skimmed milk and then incubated overnight at 4°C with specific primary antibodies. Following incubation, the membranes were washed in tris-buffered saline (TBS), incubated with secondary HRP-conjugated anti-rabbit IgG antibody for 1 h with 5% skimmed milk, and again washed in TBS at room temperature. Immune complexes were detected using a Bio-Rad ChemiDoc XRS system, and the protein expression was normalized to glyceraldehyde 3 phosphate dehydrogenase (GAPDH) expression levels.

*(2) Uptake of Sorafenib in HepG2 Cells Transfected with miR-148a Mimics and Control HepG2 Cells*. The effects of incubation time (0.5-2 h) and drug concentration (5, 10, and 15 *μ*mol/L) on sorafenib uptake by transgenic and control HepG2 cells were investigated. Uptake kinetics experiments were conducted as mentioned above.

*(3) Effect of Regulating OATP1B1 Expression on the Viability and Apoptosis Rate of HepG2 Cells*. The CCK-8 method was used to measure cell viability. After drug treatment at different time points, 400 *μ*L of 10% CCK-8 solution was added to each well, and the reaction was conducted at 37°C for 1 h. The optical density (OD) of each well was read at 450 nm and 650 nm, and the final OD value was measured as OD_450_-OD_650_. The experimental results were calculated as follows: Cell survival rate (%) = experimental group (OD_450_ − OD_650_) × 100/control group (OD_450_ − OD_650_). To study the effect of a PXR inducer on the cell viability of the control and miR-148a mimic-transfected HepG2 cells, the cells were incubated with or without 5 *μ*mol/L rifampicin for 24 h. Following this, sorafenib was added, and the cells were further incubated for approximately 24 h. The CCK-8 method was used to measure cell viability. The half-maximal inhibitory concentration (IC_50_) was calculated after incubation for 24 h at 37°C and 5% CO2. To study the effects of sorafenib on the cell cycle of transgenic HepG2 cells, we used flow cytometry for different cycles after the cells had been incubated for approximately 36 h.

#### 2.3.3. Pharmacokinetic Changes of Sorafenib after PXR Regulation of Oatp2 Expression and Its Effect on the Therapeutic Effect of Liver Cancer in Rats

*(1) Establishment of PXR-Activated Liver Cancer Rat Model*. The Solt-Farber method of cancer induction was applied to promote diethylnitrosamine- (DEN-) induced liver cancer in rats. The rats were intraperitoneally injected with 200 mg/kg DEN solution at one time and then fed with a diet containing 0.02% 2-acetaminofluorene for 14 days after 2 weeks. For most of the rats, liver resections were performed in the third week, and normal diet resumed in the fourth week. After 4 weeks, the pathological sections were observed to detect liver cancer foci in the rat liver. Following this, the PCN activation method was used to construct the rat model. Rats in the experimental group were intraperitoneally injected with PCN (75 mg/kg) for 4 consecutive days, whereas those in the control group were intraperitoneally injected with a similar volume of normal saline. The rats were killed 24 h after the last administration, and the Oatp2 expression was detected by RT-qPCR testing and Western Blotting.

*(3) Pharmacokinetic Analysis (*n* = 5)*. Ten rats with liver cancer were divided into two groups: the experimental group (*n* = 5, the rats were intraperitoneally injected with PCN (75 mg/kg) for 4 consecutive days) and the control group (*n* = 5, not treated with PCN). All rats in the two groups received gavage administrations of sorafenib (50 mg/kg). Before 0 and at 0.5, 1, 1.5, 2, 3, 4, 6, 8, 12, 24, 48, 72, and 96 h after sorafenib administration, 0.2 mL blood was collected from the caudal vein. All blood samples were centrifuged at 3,000 × g for 5 min, and the plasma was separated and stored at -80°C until analysis.

*(4) Evaluation of Treatment Efficacy (*n* = 10)*. Twenty rats with liver cancer were divided into two groups: the experimental group (*n* = 10, the rats were intraperitoneally injected with PCN (75 mg/kg) for 4 consecutive days) and the control group (*n* = 10, not treated with PCN). All the rats in the two groups were administered sorafenib (50 mg/kg/d for 20 days) by gavage. Following this, the serum aspartate aminotransferase (AST), alanine aminotransferase (ALT), and total bilirubin (TB) levels in rats were measured. The peritumoral tissues were pathologically examined, and the microvascular density (MVD) and levels of vascular endothelial growth factor (VEGF) were measured (*n* = 5). The main procedures of immunohistochemistry were as follows: xylene dewaxing, gradient alcohol rehydration; blocking and inactivating endogenous peroxidase; antigen repair; primary antibody were added and incubated overnight in a refrigerator at 4°C, and then, the temperature was turned to room temperature for equilibrium for 30 min, and PBS was rinsed for 3 × 5 min; the secondary antibody was added and then incubated at 37°C for 30 min and rinsed with PBS for 3 × 5 min; DAB staining, reaction progress was observed under a microscope, and the reaction was thoroughly rinsed with tap water; lignin redyeing, drying, sealing, and photographing; VEGF positive rate and MVD count. Survival time and survival rate of the sorafenib-treated and control groups were observed for 60 days (*n* = 5).

### 2.4. Statistical Analysis

All data are presented as the mean ± SE. Statistical analysis was performed using GraphPad Prism 5.0 (GraphPad Software Inc., San Diego, CA, USA). All *t*-tests were two-tailed, and *P* < 0.05 was considered statistically significant. To determine the pharmacokinetics of sorafenib, the concentration-time data were analyzed using the Drug and Statistics software (DAS version 2.0, Center of Institute of Clinical Pharmacology, Nanchang University, Nanchang, Jiangxi, China).

## 3. Results

### 3.1. Effect of OATP1B1 Genetic Mutations on Drug Transport and Treatment Effect in HepG2 and LO2 Cells

**Figure 1:**
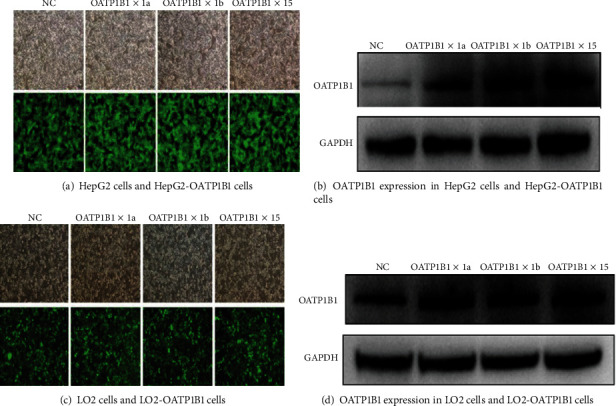
OATP1B1 expression in control and transgenic HepG2 and LO2 cells. The fluorescent photos show HepG2 and LO2 control cells and the cells after being transfected with lentivirus containing pGC-FU-OATP1B1∗1a, pGC-FU-OATP1B1∗1b, and pGC-FU-OATP1B1∗15 (a, c). OATP1B1 expression is detected by Western Blotting and RT-qPCR. Compared to control GFP-HepG2 cells, OATP1B1 expression increases to approximately 110.41%, 147.92%, and 193.75% in OATP1B1∗1a-GFP-HepG2, OATP1B1∗1b-GFP-HepG2, and OATP1B1∗15-GFP-HepG2 cells, respectively (b). Compared to control GFP-LO2 cells, OATP1B1 expression increases to approximately 32.93%, 45.12%, and 35.37% in OATP1B1∗1a-GFP-LO2, OATP1B1∗1b-GFP-LO2, and OATP1B1∗15-GFP-LO2 cells, respectively (d). Protein level of OATP1B1 in HepG2 than LO2 cells was not obvious difference. NC: control.

We successfully established transgenic cell models of OATP1B1∗1a-HepG2, OATP1B1∗1b-HepG2, OATP1B1∗15-HepG2, OATP1B1∗1a-LO2, OATP1B1∗1b-LO2, and OATP1B1∗15-LO2. OATP1B1 was highly expressed in these HepG2 and LO2 cells after being transfected with the OATP1B1-lentivirus plasmid. Western Blot and RT-qPCR test results indicated that the expression of OATP1B1∗15 was higher in OATP1B1∗15-HepG2 cells than in OATP1B1∗1a-HepG2 cells (Figure 1). RT-qPCR test results show higher mRNA expression in transgenic HepG2 cells than in control cells (0.00 ± 0.00 vs. 1.13 ± 0.42 vs. 1.29 ± 0.22 vs. 1.00 ± 0.081, respectively). Compared to control GFP-LO2 cells, OATP1B1 expression increases to approximately 32.93%, 45.12%, and 35.37% in OATP1B1∗1a-GFP-LO2, OATP1B1∗1b-GFP-LO2, and OATP1B1∗15-GFP-LO2 cells, respectively. RT-qPCR test results also show higher mRNA expression in transgenic LO2 cells than in control cells (0.00 ± 0.00 vs. 1.25 ± 0.43 vs. 1.80 ± 0.48 vs. 0.73 ± 0.32, respectively). OATP1B1 expression in HepG2 cells is higher than in LO2 cells but showed no significantly difference.

**Figure 2:**
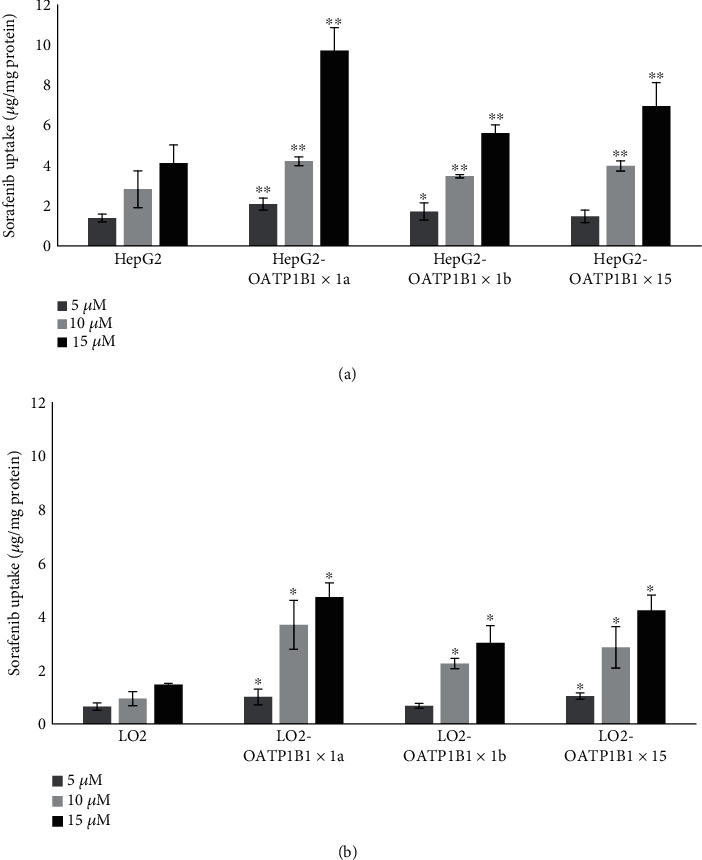
Uptake of sorafenib in control and transgenic HepG2 and LO2 cells. HepG2, OATP1B1-HepG2, LO2, and OATP1B1-LO2 cells were incubated with different concentrations of sorafenib (5, 10, and 15 *μ*M) for 10 min (a, b). Overexpression of OATP1B1 in HepG2 cells significantly increases the uptake of sorafenib compared with control of HepG2 cells (a). Gene mutations significantly decrease the intake of sorafenib in OATP1B1∗1b-HepG2 cells (decrease by about 17.77% and 28.47%) and OATP1B1∗15-HepG2 cells (decrease by about 42.24% and 28.47%) compared to those of OATP1B1∗1a-GFP-HepG2 cells when treated with sorafenib concentrations of 10 and 15 *μ*M, respectively (a). Similar to the patterns in OATP1B1-HepG2 cells, OATP1B1 overexpression in LO2 cells significantly increases sorafenib uptake of sorafenib compared with control of LO2 cells (b). Gene mutations also decrease the intake of sorafenib in OATP1B1∗1b-GFP-LO2 cells (decrease by about 39.02% and 22.76%) and OATP1B1∗15-GFP-LO2 cells (decrease by about 35.94% and 10.57%) compared to those of OATP1B1∗1a-LO2 cells when treated with sorafenib. Concentrations of 10 and 15 *μ*M, respectively (b). Interestingly, sorafenib uptake in HepG2 and OATP1B1-HepG2 cells are all significantly higher than in LO2 and OATP1B1∗1a-LO2 cells (a, b). ^∗^*P* < 0.05; ^∗∗^*P* < 0.01.

We initially studied the uptake pharmacokinetics features of OATP1B1-mediated transport of sorafenib by measuring cellular intake in OATP1B1-overexpressing HepG2 and LO2 cells. As shown in Figures 2(a) and 2(b), overexpression of OATP1B1 could significantly increases the uptake of sorafenib in transgenic cells of HepG2 and LO2. The intake of sorafenib in OATP1B1∗1a-HepG2 was significantly higher than HepG2 cells and increased by about 49.64, 50.00, and 136.16%, respectively, when the concentrations of sorafenib were 5, 10, and 15 *μ*mol/L. The same results also showed in OATP1B1∗1a-LO2 cells and increased by about 56.25, 292.55, and 223.97%. Among the different gene types, OATP1B1∗1a-HepG2 and OATP1B1∗1a-LO2 cells showed the highest uptake (more than 1.5-fold that observed in control cells). Notably, OATP1B1 gene mutations affected the uptake of sorafenib. Compared to OATP1B1∗1a, both OATP1B1∗1b and OATP1B1∗15 reduced the uptake of sorafenib in HepG2 and LO2 transgenic cells. Simultaneously, we found that the uptake of sorafenib in HepG2 was obviously higher than in LO2 cells and increased by about 117.19, 200.00, and 182.19%, respectively, when the concentrations of sorafenib were 5, 10, and 15 *μ*mol/L. The same results could also see in transgenic cells of HepG2. As the mRNA and protein level of OATP1B1 in HepG2 than LO2 cells was not obvious difference, therefore, the reason for this uptake difference may be related with the function change of OATP1B1 in HepG2.

**Figure 3:**
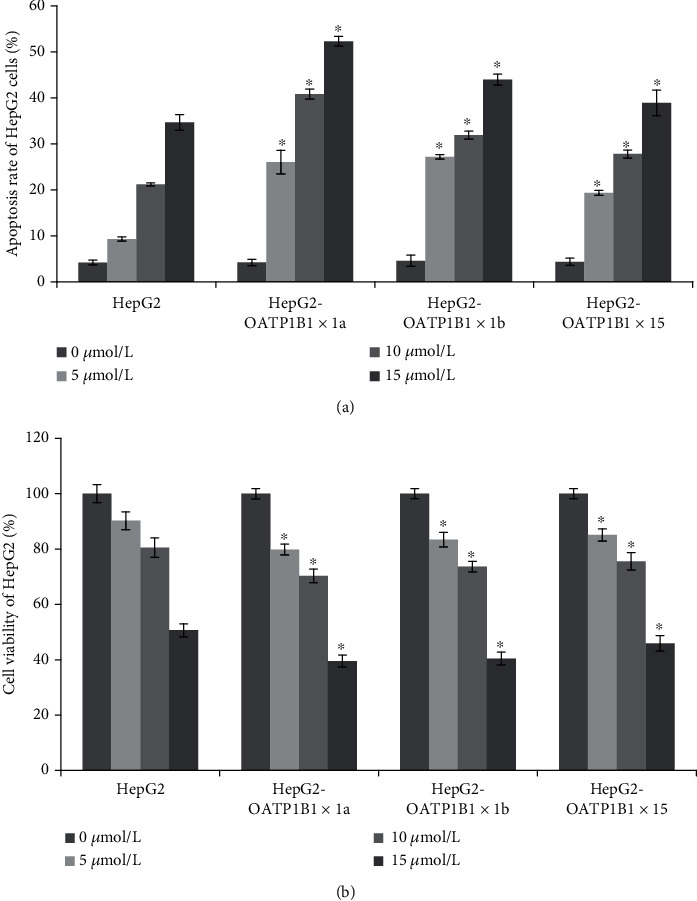
(a) Apoptosis rate of HepG2, HepG2-OATP1B1∗1a, HepG2-OATP1B1∗1b, and HepG2-OATP1B1∗15 cells incubated with sorafenib (0-15 *μ*M). After sorafenib treatment, the rate of apoptosis increased significantly in OATP1B1-HepG2 cells compared to that in the control group. Gene mutations also affected the rate of apoptosis of HepG2 cells. (b) Cell viability of HepG2, HepG2-OATP1B1∗1a, HepG2-OATP1B1∗1b, and HepG2-OATP1B1∗15 cells incubated with sorafenib (0-15 *μ*M). Cell viability in HepG2-OATP1B1∗1a, HepG2-OATP1B1∗1b, and HepG2-OATP1B1∗15 cells was significantly decreased compared with HepG2 cells (*P* < 0.05), and we found that the cell viability was higher in OATP1B1∗1b or ∗15-HepG2 cells than in OATP1B1∗1a-HepG2 cells after sorafenib treatments. ^∗^*P* < 0.05.

After sorafenib treatment, the rate of apoptosis increased significantly in OATP1B1-HepG2 cells compared to that in the control group (Figure 3(a)). When the concentrations of sorafenib were 5, 10, and 15 *μ*mol/L, the rate of apoptosis in OATP1B1∗1a-HepG2 increased by about 78.04, 92.63, and 50.95% compared with HepG2 cells. Gene mutations also affected the rate of apoptosis of HepG2 cells; for instance, the rate of apoptosis in OATP1B1∗1b and OATP1B1∗15-HepG2 cells was lower than that of OATP1B1∗1a-HepG2 cells (Figure 3(a)). Correspondingly, cell viability in OATP1B1∗1a-HepG2 was decreased about 12.62, 12.67, and 21.97% compared with HepG2 cells, and we found that the cell viability was higher in OATP1B1∗1b and OATP1B1∗15-HepG2 cells than in OATP1B1∗1a-HepG2 cells after sorafenib treatments (5-15 *μ*mol/L) (Figure 3(b)).

### 3.2. Effect of Regulating OATP1B1 Expression on the Viability and Apoptosis Rate of HepG2 Cells

**Figure 4:**
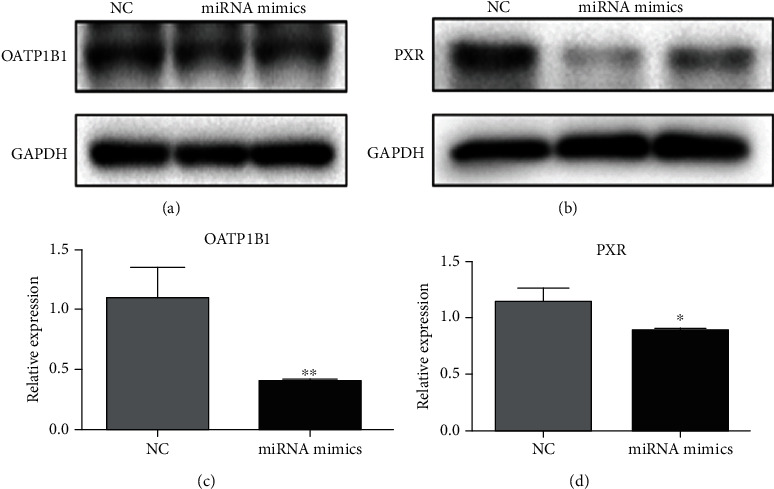
Protein and mRNA expression levels of OATP1B1 and PXR in HepG2 cells following transfection with miR-148a mimics. Compared to control cells, OATP1B1 expression is significantly lower in transfected cells (0.90 ± 0.02 vs. 0.81 ± 0.06) (a). Similar to protein expression, mRNA levels of OATP1B1 are significantly lower in transfected cells (1.10 ± 0.26 vs. 0.41 ± 0.01) (c). Expression of PXR proteins in HepG2 cells is significantly lower after transfection with miR-148a mimics (0.84 ± 0.13 vs. 0.32 ± 0.08) (b). mRNA levels of PXR are also significantly lower than in control cells (1.15 ± 0.12 vs. 0.90 ± 0.01) (d). NC: control. ^∗^*P* < 0.05; ^∗∗^*P* < 0.01.

**Figure 5:**
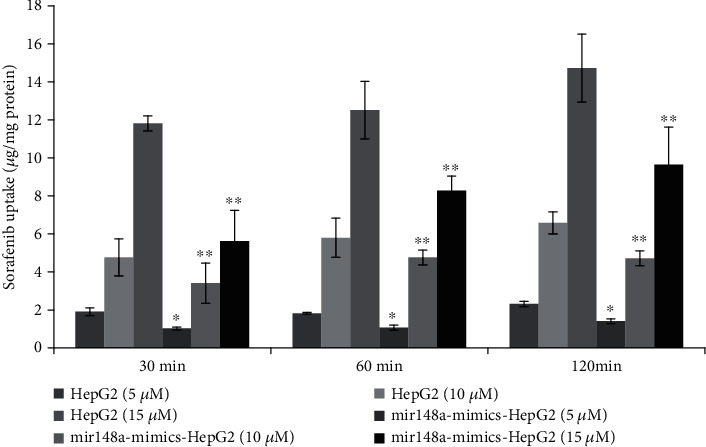
miR-148a affected the uptake of sorafenib in HepG2 cells incubated for different time (30-120 min) with varying concentrations of sorafenib (5-15 *μ*mol/L). Bars represent the mean ± SE (*n* = 3) of sorafenib uptake in control and transfected HepG2 cells incubated with different concentrations of sorafenib (5, 10, and 15 *μ*M) for 30, 60, and 120 min, respectively. Intake of sorafenib increased significantly with concentration increasing in both groups of cells. Transfection with miR-148a mimics significantly affects the uptake of sorafenib in HepG2 cells (^∗^*P* < 0.05; ^∗∗^*P* < 0.01). However, incubation time has no apparent effect on the uptake of sorafenib in control and transfected HepG2 cells. ^∗^*P* < 0.05; ^∗∗^*P* < 0.01.

When miR-148a mimics were transfected into HepG2 cell lines, OATP1B1 and PXR expression levels decreased significantly (Figure 4), indicating that miR-148a inhibits the expression of these proteins. Simultaneously, miR-148a affected the uptake of sorafenib in HepG2 cells incubated for different time (30-120 min) with varying concentrations of sorafenib (5-15 *μ*mol/L) (Figure 5). As shown in Figure 5, when the cells were incubated approximately 30 min, intracellular sorafenib accumulation was reduced by 46.1%, 28.5%, and 52.5% in cells overexpressing miR-148a after addition of 5, 10, and 15 *μ*mol/L of sorafenib, respectively. Correspondingly, the viability of HepG2 cells increased by about 8.2%, 12.1%, and 19.9%.

**Figure 6:**
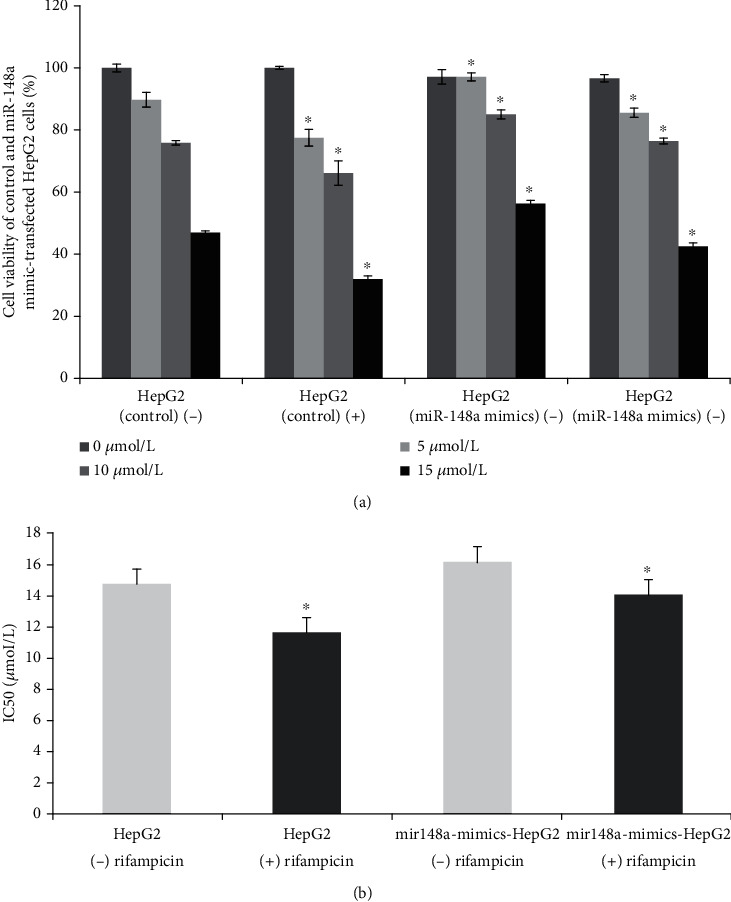
(a) Cell viability of HepG2 (control) and miR-148a mimic-transfected HepG2 cells after incubation with sorafenib (0-15 *μ*M) followed by or not by rifampicin (5 *μ*mol/L). The cell viability was significantly decreased in miR-148a mimic-transfected HepG2 cells compared with the HepG2 cells (^∗^*P* < 0.05). On adding the PXR inducer rifampicin, sorafenib significantly decreased the vitality both in HepG2 cell lines and HepG2 cell transfected with miR-148a mimics (^∗^*P* < 0.05). (b) The IC_50_ values for sorafenib in HepG2 and miR-148a mimic-transfected HepG2 cells at the experiment condition with or without rifampicin were calculated. Rifampicin is an inducer of PXR; (–) not treat with rifampicin; (+) treat with rifampicin. (^∗^*P* < 0.05).

Interestingly, after transfection with miR-148a mimics, the HepG2 cell survival rates increased by about 8.20%, 12.08%, and 19.90% when the concentrations of sorafenib were 5, 10, and 15 *μ*mol/L (Figure 6(a)). The results mean that drug's inhibitory effect on cell growth may weaken in transfected cells compared to that in the control cells. Therefore, these results confirmed that miR-148a mimics significantly affected the function of OATP1B1 in HepG2 cells; in other words, they may decrease the cancer suppression effect of sorafenib. On adding the PXR inducer rifampicin, sorafenib significantly decreased the vitality of HepG2. This phenomenon was observed in both HepG2 cell lines and HepG2 cell transfected with miR-148a mimics. Rifampicin, acts as activation of receptor, the activation of PXR may increase the expression of OATP1B1, leading to an increase in sorafenib uptake which resulting in a decline in cell vitality. Because miR-148a mimics could decrease the expression of OATP1B1, we could note that the cell viability be higher in transfected HepG2 cells than in the control group of HepG2 cells after sorafenib being added with or without rifampicin. The IC_50_ values for sorafenib in HepG2 and miR-148a mimic-transfected HepG2 cells at the experiment condition with or without rifampicin were calculated as follows (shown in Figure 6(b)): HepG2 cells without rifampicin, 14.66 ± 2.35 *μ*mol/L; HepG2 with rifampicin, 11.6 ± 1.69 *μ*mol/L; HepG2 cells transfected with miR-148a mimics without rifampicin, 16.13 ± 3.05 *μ*mol/L; and HepG2 cells transfected with miR-148a mimics with rifampicin, 14.04 ± 2.42 *μ*mol/L.

HepG2 cell cycle results showed that with increasing sorafenib concentration, the proportion of G0/G1 phase cells increased significantly; however, the number of cells in the S phase decreased significantly, and there was no significant trend in the number of G2/M phase cells (Table 1). This indicated that sorafenib has an inhibitory effect on the cell cycle and increasing drug concentration enhanced G0/G1 phase arrest. After transfection with miR-148a mimics, the proportion of HepG2 cells in G0/G1 phases decreased significantly; however, the number of cells in the S phase increased significantly, indicating that interference with the Oatp2 expression may affect the therapeutic effect of sorafenib.

### 3.3. Pharmacokinetic Changes of Sorafenib after PXR Regulation of Oatp2 Expression and Its Effect on the Therapeutic Effect of Liver Cancer in Rats

**Figure 7:**
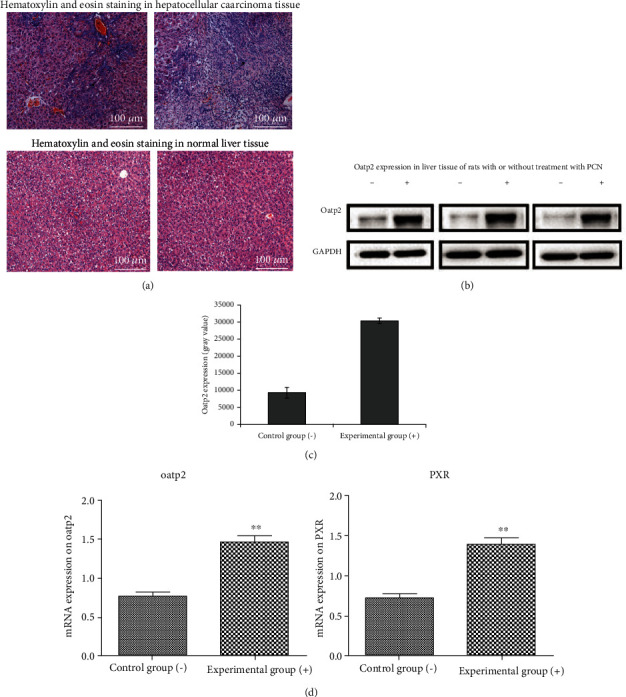
(a) Compared with hematoxylin and eosin staining in normal liver tissue, hematoxylin and eosin staining showing severe congestion in hepatocellular carcinoma tissue, scattered inflammatory cells, nuclear pyknosis in most liver nuclei, and obvious mitosis. The black arrow indicates the mitotic image. (b) OATP2 expression in liver tissue of rats with or without treatment with PCN. Liver tissues were collected and washed with ice-cold PBS 3 times and lysed with lysis buffer. Lysis buffer was collected and centrifuged at 1.4 × 10^4^ rpm for 20 min, and supernatants were used to analyze OATP2 levels by Western Blotting. Compared to the control rats, OATP2 expression is significantly increased in rats treated with PCN. (c) Western Blot bands show that OATP2 expression increases to approximately 227.7% of that in control rats. (d) mRNA expression of OATP2 and PXR in the experimental group is also significantly higher than in the control group. Symbols indicate rat models of hepatocellular carcinoma untreated (-) or treated (+) with PCN. ^∗^*P* < 0.05; ^∗∗^*P* < 0.01.

**Figure 8:**
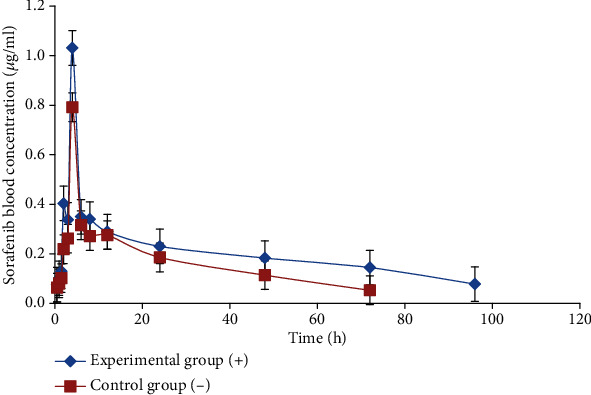
Influence of PCN (and increased OATP2 expression) on sorafenib pharmacokinetics (*n* = 5). The pharmacokinetic profiles of sorafenib in rats after oral administration of 50 mg/kg sorafenib with or without treatment with PCN (75 mg/kg) for 4 days. PCN significantly affects the pharmacokinetics of sorafenib. Symbols indicate rat models of hepatocellular carcinoma untreated (-) or treated (+) with PCN.

**Figure 9:**
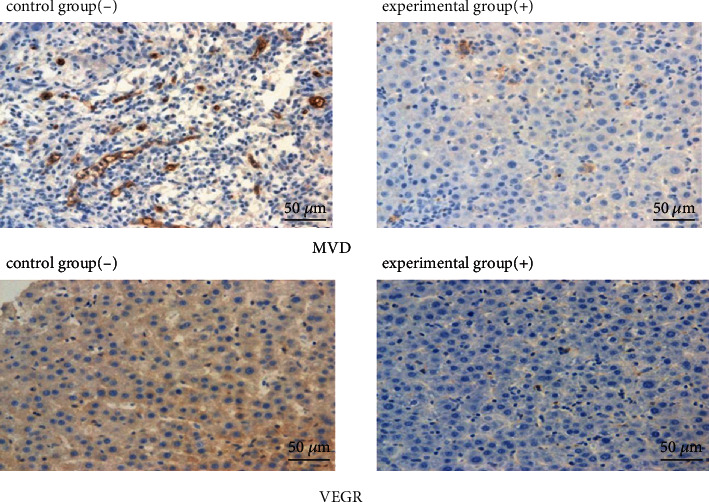
Histopathological observations of peritumoral tissues. Vascular endothelial growth factor (VEGF) levels and microvessel density (MVD) in peritumoral tissues of rats in the control and experimental groups after immunohistological staining and treatment with 50 mg/kg sorafenib for 20 days. VEGF expression and MVD counts in the experimental group were significantly lower than those in the control group (not treated with PCN). VEGF positive rate and MVD count in tumor-adjacent tissues were also significantly decreased in the experimental group (6.36 ± 2.29% vs. 18.36 ± 5.65%, 27.50 ± 11.56 vs. 53.10 ± 24.74, respectively). Symbols represent rat models of hepatocellular carcinoma untreated (-) or treated (+) with PCN.

We successfully established a rat model of hepatocellular carcinoma and found that protein or mRNA expression of both Oatp2 and PXR in the liver increased significantly after the rats being intraperitoneally injected with PCN (75 mg/kg) for 4 consecutive days (Figures 7(a)–7(d)). The blood concentration of sorafenib was determined by HPLC. Pharmacokinetic parameters were significantly different between the experimental and control groups (Figure 8, Table 2). PCN significantly affected the pharmacokinetics of sorafenib and increased its concentration in the blood and liver. Elimination half-life and the area under the concentration-time curve were significantly higher in the experimental group than in the control group and increased by about 76.28 and 73.04%, respectively. However, there was no difference in the values of ALT (U/L) (1.94 ± 0.24 vs. 1.98 ± 0.36), AST (U/L) (1000 ± 139 vs. 967 ± 150), and TB (*μ*mol/L) (2061 ± 283 vs. 2017 ± 296) in rats after oral administration of sorafenib with or without treatment with PCN. With increasing Oatp2 expression, the histopathological observations of peritumoral tissues and the degree of necrosis and degeneration of surrounding normal hepatocytes were significantly improved compared to those of the control group. VEGF positive rate and MVD count in tumor-adjacent tissues were also significantly decreased in the experimental group (6.36 ± 2.29% vs. 18.36 ± 5.65%, 27.50 ± 11.56 vs. 53.10 ± 24.74, respectively, *P* < 0.05) (Figure 9). Finally, the survival time and survival rate of the sorafenib treatment group and control group were observed for 60 days. The results showed that survival-related traits improved after the Oatp2 expression being increased due to the PCN-mediated activation of PXR (survival rate 90% vs. 80%).

## 4. Discussion

In this study, we demonstrated that sorafenib was a substrate for the human OATP1B1 transporter. Using HepG2 and LO2 cell models overexpressing the OATP1B1-type proteins, we determined that sorafenib was incorporated into cells in a concentration-dependent manner and found that the cell intakes in HepG2 were significantly higher than in LO2. Furthermore, sorafenib transport was lower in cells expressing the naturally occurring OATP1B1 variants (OATP1B1∗1b and OATP1B1∗15) compared with wild type of OATP1B1∗1a, thus, exhibiting reduced transport function. Polymorphisms of OATP1B1 could significantly affect the uptake kinetics of sorafenib both in HepG2 and LO2 cell.

Overexpression of OATP1B1 in HepG2 also affecting the pharmacological effect of sorafenib, which showed that the rate of apoptosis increased significantly in OATP1B1-HepG2 cells compared to that in the control group, correspondingly, cell viability was significantly reduced. Interesting, the rate of apoptosis of HepG2-OATP1B1∗1a cells was significantly higher than those of HepG2-OATP1B1∗1b and HepG2-OATP1B1∗15 cells when the concentrations of sorafenib were 10 and 15 *μ*M. Polymorphisms of OATP1B1 significantly affected the treatment effect of sorafenib in hepatocellular carcinoma, consistent with the notion that certain reduced function variants of *SLCO1B1* (the gene encoding OATP1B1) were associated with an increased risk of sorafenib-associated toxicity [11]. These results suggest that OATP1B1 may play an important role in pharmacodynamics of sorafenib.

We also found that miR-148a mimics reduced the protein and mRNA expression levels of OATP1B1 and PXR in HepG2 cells. Simultaneously, after being incubated about 30 min, intracellular sorafenib accumulation was reduced by 46.1%, 28.5%, and 52.5% in cells overexpressing miR-148a after the addition of 5, 10, and 15 *μ*mol/L of sorafenib, respectively. Correspondingly, the viability of HepG2 cells increased by about 8.2%, 12.1%, and 19.9%. Simultaneously, when the PXR activator rifampicin was added, the viability of both HepG2 and miR-148a mimic-transfected HepG2 cells was decreased; however, the cell viability of the transfected cells was still higher than that of HepG2 cells. The activation of PXR may increase the expression of OATP1B1, leading to an increase in sorafenib uptake.

A previous study showed that PCN increased the expression of both Mdr1a/1b mRNA and P-gp protein in the intestine and brain. PCN also increases the expression of Mdr1a/1b mRNA in the liver [12]. Another in vivo study showed that in rats pretreated with verapamil, the *C*_max_ of sorafenib increased by about 57.40% from 55.73 ng/mL to 87.72 ng/mL, and the area under the curve (AUC) _(0-t)_ increased by approximately 58.2% when sorafenib was coadministered with verapamil. These results indicate that P-gp is involved in the transport of sorafenib, and verapamil acts as a P-gp inhibitor that could increase its absorption [13]. Therefore, when the PCN is combined with sorafenib, the absorption of sorafenib may decrease. However, the results of our study are contrary to this hypothesis. Our results showed that when the rats were pretreated with PCN, the plasma concentration of sorafenib was significantly increased. Compared to that in the control rats, Oatp2 protein expression was significantly higher after the hepatoma mice were treated with PCN for 4 days. We hypothesize that after pretreatment with PCN, a significant increase in the OATP2 expression in liver tissue or the intestine would increase the uptake of sorafenib, which is beyond the P-gp-mediated efflux; this is a noteworthy and important finding. Previous research has shown that there were minimal differences in peak plasma concentration and plasma AUC for sorafenib, sorafenib N-oxide, and total active compounds (sorafenib + sorafenib N-oxide) between OATP1b2(-/-) and wild-type mice after a single oral sorafenib dose of 10 mg/kg [3]. However, the results of our study are contrary to the findings of this study. This may be due to differences in the expression of OATPs in hepatocellular carcinoma rats and mice, which may result in changes in the expression and function of OATPs under conditions of liver cancer. Although the survival rate of hepatocellular carcinoma rats improved after the Oatp2 expression being increased, this result showed no statistical difference, and larger sample sizes of liver cancer rats are needed to explore whether survival can be improved.

Sorafenib is also reported to be metabolized by CYP3A4 in the liver; a study showed that triptolide might also cause a higher *C*_max_ and lower oral clearance rate of sorafenib by inhibiting CYP3A-mediated metabolism [14]. Further research is needed to elucidate whether PCN changes the expression and function of CYP3A4 in a rat model of liver cancer and whether it affects the pharmacokinetics and pharmacodynamics of sorafenib.

## 5. Conclusions

OATP1B1 plays an important role in the pharmacokinetics and pharmacodynamics of sorafenib. Changes in the expression and function of OATP1B1 would significantly affect the uptake of sorafenib in HepG2 and LO2 transgenic cells. The uptake of sorafenib by HepG2 cells was higher than that by LO2 cells. Simultaneously, the uptake of sorafenib in HepG2 was significantly higher than in miR-148a mimic-transfected HepG2 cells. These results confirmed that miR-148a mimics would significantly affect the function of OATP1B1 in HepG2 cells; in other words, they may decrease the cancer suppression effect of sorafenib. PCN could significantly increase the expression of Oatp2 and affect the pharmacokinetics of sorafenib. At the same time, VEGF levels and MVD in tumor-adjacent tissues decreased significantly, suggesting that increased Oatp2 expression improves the treatment effect of sorafenib in a rat model of liver cancer [15].

## Figures and Tables

**Table 1:** Effect of different concentrations of sorafenib (0-15 *μ*M) treatment on cell cycles of miR-148a mimic-transfected and control HepG2 cells.

Cycles	Cells	0 *μ*mol/L	5 *μ*mol/L	10 *μ*mol/L	15 *μ*mol/L
G1	HepG2	56.27 ± 0.25	58.57 ± 1.01	62.83 ± 0.57	67.20 ± 0.46
	HepG2 (miR-148a)	54.67 ± 0.78	54.87 ± 0.35	60.77 ± 0.68	62.93 ± 0.93
S	HepG2	31.70 ± 0.50	26.57 ± 3.80	24.50 ± 3.80	21.13 ± 0.96
	HepG2 (miR-148a)	31.57 ± 1.51	33.00 ± 0.44	26.90 ± 2.03	24.03 ± 1.65
G2	HepG2	10.46 ± 0.66	12.43 ± 2.07	13.26 ± 4.53	11.70 ± 0.98
	HepG2 (miR-148a)	9.36 ± 1.78	10.26 ± 0.43	13.37 ± 1.96	11.23 ± 0.68

**Table 2:** Pharmacokinetic parameters of sorafenib in rats after oral administration of sorafenib with or without PCN.

Parameters	(-) PCN treatment	(+) PCN treatment
K*α* (h^−1^)	0.98 ± 0.17	0.79 ± 0.11
*α* (h^−1^)	0.67 ± 0.15	0.78 ± 0.14
*β* (h^−1^)	0.026 ± 0.013	0.016 ± 0.003
Tka (h)	0.69 ± 0.15	0.88 ± 1.12
T*α* (h)	1.03 ± 0.35	0.89 ± 0.16
T*β* (h)	27.07 ± 4.38	47.72 ± 4.61^∗^
AUC (*μ*g/mL/h)	125.55 ± 11.11	217.26 ± 26.42^∗^

(+) PCN treatment: sorafenib (50 mg/kg) was administered orally with PCN treatment (75 mg/kg); (-) PCN treatment: sorafenib (50 mg/kg) was administered orally without PCN treatment (75 mg/kg); K*α* (h^−1^): absorption rate constant; *α* (h^−1^): distribution rate constant; *β* (h^−1^): elimination rate constant; Tka (h): absorption half-life; T*α*: distribution half-life; T*β* (h): elimination half-life; AUC (*μ*g/mL/h): area under concentration-time curve. (^∗^*P* < 0.05 compared with (-) PCN treatment).

## Data Availability

The data used during the study are available online https://osf.io/z3aq4/?view_only=a1f302519f8647509a658d114f292364. The authors can also make data available on request through an email to the corresponding author, wenjh8606@163.com, Prof. Dr. Wen.

## Abbreviations

ALT: Alanine aminotransferase
AST: Aspartate aminotransferase
GAPDH: Glyceraldehyde 3 phosphate dehydrogenase
HPLC: High performance liquid chromatography
MVD: Microvascular density
OATP1B1: Organic anion transport polypeptide 1B1
PCN: Pregnenolone-16*α*-carbonitrile
PVDF: Polyvinylidene difluoride
PXR: Pregnane X receptor
RT-qPCR: Real-Time Quantitative reverse transcription PCR
SDS-PAGE: Sodium dodecyl sulfate polyacrylamide gel electrophoresis
TB: Total bilirubin
VEGF: Vascular endothelial growth factor.
